# Do Earthquakes Shake Stock Markets?

**DOI:** 10.1371/journal.pone.0133319

**Published:** 2015-07-21

**Authors:** Susana Ferreira, Berna Karali

**Affiliations:** Department of Agricultural and Applied Economics, The University of Georgia, Athens, Georgia, United States of America; Feng Chia University, TAIWAN

## Abstract

This paper examines how major earthquakes affected the returns and volatility of aggregate stock market indices in thirty-five financial markets over the last twenty years. Results show that global financial markets are resilient to shocks caused by earthquakes even if these are domestic. Our analysis reveals that, in a few instances, some macroeconomic variables and earthquake characteristics (gross domestic product per capita, trade openness, bilateral trade flows, earthquake magnitude, a tsunami indicator, distance to the epicenter, and number of fatalities) mediate the impact of earthquakes on stock market returns, resulting in a zero net effect. However, the influence of these variables is market-specific, indicating no systematic pattern across global capital markets. Results also demonstrate that stock market volatility is unaffected by earthquakes, except for Japan.

## Introduction

Over the past few decades the world has witnessed an increase in the reported frequency and damages caused by natural disasters, particularly hydro-meteorological disasters [1,2,3]. A growing literature has begun to analyze their economic and broader socio-political impacts. The majority of economic studies evaluate the short- and long-run impact of natural disasters on macroeconomic indicators, primarily gross domestic product (GDP) and its annual growth, typically using panel vector auto regressions or growth regressions [4,5,6,7,8,9]. While the findings are sometimes inconclusive, the general consensus is that disasters do not always negatively impact GDP growth or long-term GDP but that when they do, the negative impacts are larger for developing countries. Fomby et al. [10] demonstrate that it is very important to account for disaster type. In their study, droughts have a negative impact on GDP growth, whereas moderate floods have a positive impact and earthquakes have no effect. Similarly, Raddatz [6] finds an insignificant effect of geological disasters (a category that includes earthquakes) on GDP per capita, while Skidmore and Toya [4] show that geological events depress long-run GDP growth.

In this paper, we use an alternative approach to estimate the aggregate economic impacts of natural disasters. We analyze whether earthquakes are capitalized into global stock markets. Unlike other natural disasters (e.g. droughts or even floods and storms), earthquakes have a very rapid onset that is arguably a surprise to stock markets. That is, their timing is exogenous. In addition, earthquakes can be very destructive. For example, Cavallo et al. [11] estimate the direct cost of the earthquake that struck Haiti in January 2010 and killed about 2.4% of its population at US$8.1 billion (more than 100% of the value of Haitian GDP, and yet a figure that is considered to be a lower-bound estimate). The costliest natural disaster on record is the 2011 Tohoku-Oki Earthquake and Tsunami, whose direct damages are estimated at US$211 billion [12]. Earthquakes can also have a marked impact in countries other than the one where the epicenter is located, for example in neighbors or trading partners. Disruptions in the supply chains in the US car and consumer electronic industries, and dramatic energy policy changes in Germany following the 2011 Tohoku-Oki event in Japan suggest that considering the response of global capital markets to natural disasters, earthquakes in particular, in an increasingly connected world is potentially important.

The literature analyzing the impact of natural disasters on capital markets is scarce and consists mainly of case and event studies estimating the impact of “domestic” disasters on specific sectors such as insurance, construction, and real estate [13,14,15,16,17,18]. A number of studies have analyzed “contagion” effects in international financial markets, for example following the Mexican peso collapse in 1994, the East-Asian crisis in 1997–1998, and the Russian crisis in 1998 [19,20,21]. Applied to natural disasters, we are aware of only one study, Lee et al. [22], analyzing the contagion effect across international financial markets one to three months after the South-East Asia Tsunami on December 26, 2004.

Different from earlier studies, our paper is not limited to domestic earthquakes; we analyze the impact of the largest 24 earthquakes that happened over the last two decades across the world on the returns to the aggregate stock market indices of 35 different financial markets (all the markets for which complete data were available). Further, we analyze the immediate, not the prolonged impact of those large earthquakes on stock markets. Provided stock markets are relatively efficient, the impact of earthquakes should be reflected in short-run stock price changes. These price changes signal market beliefs about expected changes in future profitability arising from the occurrence of the disaster. Hatase et al. [23] find evidence of increased exchange rate volatility in the period following three great earthquakes in Japan, a phenomenon that is also observed in other countries. They hypothesize that this excess volatility following major (domestic) earthquakes reflects the fact that a devastating earthquake increases uncertainty about the nation’s future economic fundamentals.

In contrast, our focus in this paper is on aggregate stock market performance. We use aggregate market indices calculated from data on a representative sample of stocks covering a minimum of 75 to 80 percent of total market capitalization in each market. Provided that market indices are a fair representation of the future prospects of overall (rather than sector-specific) economic performance, our approach can then be considered complementary to macroeconomic studies focusing on the impacts of earthquakes on GDP or GDP growth. We note, however, that the comparison can only be approximate, since, by construction, market indices exclude non-publicly traded companies. These can represent a sizeable proportion of the total economic activity, especially in countries with large informal sectors.

In a recent paper, Scholtens and Voorhorst [24] report the cumulative average abnormal returns of 19 stock market indices following 101 domestic earthquakes. In contrast, we analyze the impacts of large earthquakes in all the stock markets for which data were available. Moreover, one key contribution of our paper is that we analyze the impact of large earthquakes on both the conditional mean and conditional variance of stock market returns. We estimate a GARCH-X(1,1) model for each of the 35 financial markets, to investigate the impact of earthquakes on abnormal returns and on stock market volatility. Another important contribution of our paper is that we explicitly address the heterogeneity of impacts of the “average” large earthquake in different financial markets and investigate the channels through which stock markets may capitalize earthquake shocks. That is, we take into account a number of factors that may mitigate or exacerbate earthquake impacts on returns. We classify these factors into two broad categories. The first category includes indicators of the proximity between financial markets and earthquake locations (trade linkages between the country experiencing the earthquake and the country/region where the financial market is located, and geographical distance between the epicenter of the earthquake and the financial market). The second category includes indicators of the potential vulnerability and exposure of the economy to shocks (GDP per capita and trade openness) in both earthquake and financial market countries/regions. We also control for earthquake characteristics (magnitude, death toll, and affected population) and for whether the earthquake resulted in a tsunami.

We find no systematic effect of earthquakes on the returns of aggregate stock market indices, which suggests that international financial markets are resilient to large earthquake shocks. On average, some macroeconomic variables (notably GDP and trade openness) and earthquake characteristics (magnitude, whether it resulted in a tsunami, distance to the epicenter, and fatalities) are found to mediate the impact of earthquakes on abnormal returns, but in the very few cases in which these variables are found to be statistically significant their influence is highly market-specific. Reminiscent of the finding of Hatase et al. [23] for exchange rates, our results indicate that earthquakes did increase the volatility of the stock market in Japan, but not of other markets.

## Methodology

The event study methodology of Fama et al. [25] has been extensively used in the finance literature to measure stock price reactions to macroeconomic or company-related announcements as well as to any unexpected events such as terrorist attacks. There are two different commonly used methods in the literature to assess an event’s impact on stock prices [26,27]. The first method involves estimating the market model, in which company stock returns are regressed on the returns of a market portfolio, such as S&P 500, to measure the “normal” return before the event. These results are used to predict the company’s expected returns on a given day as a function of the overall market performance on that day. The difference between these predicted (normal) returns and the realized, ex-post returns during the event window are referred to as “abnormal” returns and attributed to the event. One disadvantage of this method, however, is that abnormal returns cannot be distinguished in the case of multiple or overlapping events.

The second method involves modeling abnormal returns as regression coefficients. In this case, a dummy variable taking the value of one during the event period (and zero otherwise) is added to the market model. The coefficient of the dummy variable becomes a measure of the abnormal return. One advantage of this method is that abnormal returns can be modeled as a function of observable variables. Another advantage is that multiple events can be studied simultaneously either by creating separate dummy variables for each event, or by creating a single dummy variable that equals one during each event period. In the latter case, the coefficient on the single dummy variable represents the average abnormal return across all the event periods. These two characteristics make the regression-based event study methodology particularly suitable for our study. We thus follow this regression-based event study methodology and create a single dummy variable to represent all the 24 large earthquakes considered and measure financial markets’ average abnormal return due to earthquake events. That is, using data on each of the 35 financial markets over time we estimate mean responses in stock markets to large earthquakes. While losing earthquake specificity by not introducing 24 event dummies, this method allows describing basic patterns of resilience of stock markets in a sensible and robust manner. In addition, from a policy standpoint, the relevant question is arguably not the identity of the earthquake but its nature, characterized by factors such as its location, magnitude, and damages, which, as described below, are controlled for in our model.

Daily financial asset returns have been shown to exhibit serial correlation and time-varying variance; both of which should be accounted for to obtain efficient parameter estimates [28,29]. Engle [30] developed an autoregressive conditional heteroskedasticity (ARCH) model, in which the current conditional variance depends on past values of the squared random error term. The ARCH model was modified by Bollerslev [31,32] to allow the current conditional variance to depend on the past conditional variances as well as the past squared random errors in a generalized autoregressive conditional heteroskedasticity (GARCH) model. Since then, various extensions of the GARCH model have been widely used in the literature and are found to be more appropriate in modeling stock returns [32,33,34,35].

Of particular interest to our application, GARCH-X models [36,37] allow the conditional variance to depend on additional explanatory variables. We adopt a GARCH-X(1,1) model with a Student-t error term [38,39] and allow both the conditional mean and conditional variance equations to be a function of the earthquake dummy variable. The dummy variable in the mean equation measures the abnormal returns due to earthquake events, which is the main objective of this paper, while the dummy variable in the variance equation measures the impact of the earthquakes on stock market volatility. As mentioned in the introduction, Hatase et al. [23] find that the volatility of exchange rates increases after major earthquakes, suggesting that earthquakes may increase the volatility of aggregate indices as well.

In addition to estimating abnormal market returns, we are particularly interested in shedding light on the potential factors that explain the resilience of financial markets to earthquake shocks. Thus, our model contains control variables representing transmission channels interacted with the earthquake dummy variable in the conditional mean equation of the GARCH system. Specifically, we estimate the following equation for each financial market:

$$R_{it}=\mu_{i}+\gamma_{i}d_{jt}+\pi_{i}d_{jt}I_{k_{j}=i}+\left(\psi_{i}F_{i}+\lambda_{i}H_{k_{j}}+\phi_{i}G_{ik_{j}}+\theta_{i}E_{j}+\delta_{i}D_{ij}\right)d_{jt}+\overset{5}{\underset{\tau =1}{\sum }}\zeta_{i\tau }R_{i,t-\tau }+\;\sum_{\ell =1995}^{2013}\chi_{i\ell }Y_{\ell }+\varepsilon_{it},$$ (1a)

$$\varepsilon_{it}\;\sim \;t\left(0,\;\sigma_{it}^{2},\;\nu \right),$$ (1b)

$$\sigma_{it}^{2}=\text{exp}\left(\omega_{i}+\varphi_{i}d_{jt}+\eta_{i}d_{jt}I_{k_{j}=i}\right)+\alpha_{i}\varepsilon_{\;i,t-1}^{2}+\beta_{i}\sigma_{i,t-1}^{2}$$ (1c)

for *i* = 1,…,35 financial markets; *j* = 1,…,24 earthquakes that happened in *k*_*j*_ = 1,…,15 different countries; and *t* = 1,…,5,072, covering the period of 03/02/1994–08/08/2013.

Eq (1a) is the conditional mean equation for daily returns. Eq (1b) is the error term that follows a Student-t distribution with mean zero, variance $\sigma_{it}^{2}$, and degrees of freedom *v*. Eq (1c) is the conditional variance equation, where earthquake dummy variables enter the variance specification as multiplicative heteroskedasticity [40]. Because earthquakes are concentrated along the plate boundaries of the Earth, they are more frequent in certain regions. During the period considered, there were six countries that experienced more than one large earthquake. Thus, in terms of notation, we use *j* to refer to the earthquake itself, and *k*_*j*_ to refer to the country where it happened. *R*_*it*_ is the continuously compounded daily return on stock market price index *P*_*it*_ on day *t* in financial market *i*, computed as *R*_*it*_ = 100×(*lnP*_*it*_-*lnP*_*i*,*t-1*_). We also include five lags of the dependent variable in the conditional mean equation as regressors to account for serial correlation found in daily stock returns. The variable *d*_*jt*_ is a dummy variable taking the value of one during the event window of earthquake *j*. The variable $I_{k_{j}\;=\;i}$ is an indicator variable taking the value of one if the *j* ^th^ earthquake happened where the financial market is located; that is, *k*_*j*_ = *i* (“own earthquake”). Thus, the term $d_{jt}I_{k_{j}\;=\;i}$ in (1a) accounts for the possibility of a domestic earthquake having a potentially larger impact on a given financial market. That is, the total average effect of “domestic” earthquakes is given by *γ*_*i*_+*π*_*i*_. If financial market *i* never experienced an earthquake during the sample period, then the variable $d_{jt}I_{k_{j}\;=\;i}$ drops out of the equation. As our goal is to measure the immediate impact of earthquakes we choose a relatively short window of five days following the earthquake event. Specifically, this five-day event window includes the day of the earthquake and the following five days after the earthquake to capture any resolution of initial uncertainties about the significance of the earthquake. The event window is relatively short to avoid contaminating our estimates with confounding factors following the earthquake shock that may affect stock market returns. Because the earthquake epicenter and the financial market are not necessarily (and typically are not) in the same time zone, in computing the event dummies we checked whether a given earthquake had occurred during the trading hours of each of the 35 financial markets, and account for daylight savings changes during the sample period when necessary.

The variable *F*_*i*_ includes lagged macro controls (GDP per capita and trade openness) for the financial market *i* to capture potential vulnerability to earthquake shocks, while $H_{k_{j}}$ includes the same variables for earthquake country *k*_*j*_. Income and institutional quality are widely thought to help prevent and mitigate the negative impacts of natural disasters and earthquakes in particular [41,42,43,44]. On the other hand, if an earthquake happens in a developed country, we might expect to see a larger reaction in the global financial markets than if a poor country is affected, so it is important to control for the GDP per capita of both the earthquake country and of the country where the financial market is located. Trade openness reflects the degree of economic integration. If an earthquake strikes a country without ties to the rest of the world, we would expect it to have a small impact on global financial markets. Similarly, the impact of an earthquake in a financial market located in a relatively closed region, with few ties to the rest of the world is expected to be small, *ceteris paribus*. Unlike stock market returns which are measured daily, macro variables are annual. They are lagged by one year to avoid endogeneity problems (for example, an earthquake happening today might depress this year’s output). Lagged macro variables also represent the most recent information available to traders.

$G_{ik_{j}}$ denotes lagged exports between financial market *i* and the country *k*_*j*_ in which earthquake *j* occurred to capture the strength of their economic bilateral ties. (We consider both the exports from the earthquake country to the financial market, and those from the financial market to the earthquake country). If earthquake *j* happened in financial market *i* (*k*_*j*_ = *i*), then the export variables are set to zero. *E*_*j*_ contains variables representing the characteristics of the earthquake, including its magnitude, death toll, affected population, an indicator of whether the earthquake resulted in a tsunami (such as the Tohoku-Oki Earthquake and Tsunami event in Japan). *D*_*ij*_ includes the geodetic distance between the *j* ^th^ earthquake’s epicenter and the physical location of the financial market *i* and the interaction between the distance and magnitude variables. Finally, the variable *Y*_*l*_ includes indicator variables for years 1995 through 2013 (1994 being the base year) to account for effects that are common across financial markets such as the Asian financial crisis in 1997 and the global financial crisis in 2008.

## Data

The list of major earthquakes comes from the Global Significant Earthquake Database at the National Geophysical Data Center (NGDC), a branch of the National Oceanic and Atmospheric Administration (NOAA) [45]. The NGDC database contains information on 5,833 destructive earthquakes from 2150 B.C. to the present that meet at least one of the following criteria: moderate damage (approximately US$1 million or more); 10 or more deaths; magnitude of 7.5 or greater; Modified Mercalli Intensity X or greater; the earthquake generated a tsunami. Our analysis includes the largest, most destructive earthquakes, operationalized as earthquakes that caused more than 1,000 fatalities and/or direct damages totaling more than 2.5% of the country’s GDP. To put this number into perspective, the damages of the Great Hanshin-Awaji earthquake in Japan in 1995 and the Great East Japan Earthquake in 2011 amounted to 2.71% and 3.82% of Japan’s GDP, respectively. Based on the financial market and macroeconomic data availability the sample covers 24 distinct earthquakes during the period 03/02/1994–08/08/2013.

Table 1 lists the 24 earthquakes and their key characteristics (date, location, magnitude, death toll, population affected, and damages as a percentage of GDP). The earthquake-specific control variables in Table 1 are obtained from the NGDC database and complemented with data from the U.S. Geological Survey [46] for the “official” earthquake magnitude, and the coordinates of the epicenter. Death toll represents the total number of deaths from the earthquake and secondary effects (e.g. tsunami). Geodetic distances between financial markets and earthquakes are computed using the latitude and longitude of the earthquake’s epicenter and those of the physical location of the stock markets. The number of people affected by the earthquake is computed as the population count in a 200 kilometer-radius buffer from the epicenter. We overlaid the buffer area polygons with raster population datasets for the year 1990 [47], using the isectpolyrst (Intersect Polygons with Raster) tool in the Geospatial Modelling Environment Software [48]. This tool creates summaries for each polygon based on the values in a raster layer. For our purposes, we extracted the total population count. Damages (in current US$) are obtained from NOAA and divided by the previous year’s GDP (in current US$) obtained from the World Bank’s World Development Indicators (WDI) [49]. Total numbers of earthquakes by country are listed in Table 2, in which asterisks indicate the earthquake countries that are also included as financial market countries in our sample. As seen in the table, China, Indonesia, and Iran each experienced three large earthquakes during the sample period, while El Salvador, Japan, and Russia each suffered two destructive earthquakes.

**Table 1:** List of Earthquakes.

No.	Date	Country	Magnitude	Death Toll	Damages (% of GDP)	Affected Population (200km buffer zone)
1	10/04/1994	Russia	7.3	11	2.69	111,427
2	01/16/1995	Japan	6.9	5,502	2.71	35,500,000
3	05/27/1995	Russia	7.1	1,989	0.08	179,206
4	02/28/1997	Iran	6	1,100	0.00	7,864,242
5	05/10/1997	Iran	7.2	1,728	0.09	1,303,244
6	07/17/1998	Papua New Guinea	7	2,205	0.00	257,902
7	01/25/1999	Columbia	6.1	1,185	1.89	16,400,000
8	08/17/1999	Turkey	7.6	17,118	7.43	13,900,000
9	09/20/1999	Taiwan, China	7.7	2,297	1.37	19,700,000
10	01/13/2001	El Salvador	7.7	844	5.73	7,682,112
11	01/26/2001	India	7.6	20,005	0.55	9,961,572
12	02/13/2001	El Salvador	6.5	315	2.65	9,518,647
13	05/21/2003	Algeria	6.8	2,266	8.76	9,541,817
14	12/26/2003	Iran	6.6	31,000	0.03	1,225,175
15	12/26/2004	Indonesia	9.1	227,898	4.26	941,465
16	10/08/2005	Pakistan	7.6	86,000	5.31	24,200,000
17	05/26/2006	Indonesia	6	5,749	1.08	41,300,000
18	05/12/2008	China	7.9	87,587	3.47	30,700,000
19	09/30/2009	Indonesia	7.6	1,117	0.43	4,794,427
20	01/12/2010	Haiti	7	316,000	123.64	8,384,017
21	02/27/2010	Chile	8.8	521	17.44	2,433,050
22	04/13/2010	China	6.9	2,220	0.01	389,980
23	02/21/2011	New Zealand	6.1	363	14.29	461,356
24	03/11/2011	Japan	9	15,854	3.82	5,540,704

The magnitudes reported are those which the U.S. Geological Survey (USGS) considers official for the listed earthquakes. Death toll and damages in dollar amounts are obtained from National Oceanic and Atmospheric Administration (NOAA). Death toll represents the total number of deaths from the earthquake and secondary effects. Damages are presented as a percentage of GDP obtained from World Bank's World Development Indicators (WDI). Affected population represents total number of people in a buffer zone of 200 km around earthquake's epicenter computed by authors using 1990 population survey.

**Table 2:** Number of Earthquakes by Country.

Country	No. of Earthquakes
Algeria	1
Chile*	1
China*	3
Colombia	1
El Salvador	2
Haiti	1
India	1
Indonesia*	3
Iran	3
Japan*	2
New Zealand*	1
Pakistan	1
Papua New Guinea	1
Russia	2
Turkey*	1

Asterisks (*) indicates the countries with financial markets.

Table 3 lists all the variables used in the analysis along with their descriptions, units of measurement, and data sources. Macro variables for both financial market and earthquake countries are obtained from the WDI [49]. Specifically, we include GDP per capita in constant 2005 dollars and trade openness, computed as the sum of exports and imports of goods and services and stated as a percent of GDP. Bilateral trade data are obtained from the World Bank’s World Integrated Trade Solutions (WITS) [50]. This data set contains export values of reporting countries/regions in current US$ to their trading partners. Our interest is in the bilateral ties between the country experiencing the earthquake and the country/region where the financial market is located. We state each reporting country’s export value from WITS as a percent of their total exports of goods and services obtained from WDI.

**Table 3:** Definition of Variables.

Name	Definition	Unit	Source
Returns	Daily compounded returns on stock market price index	%	Datastream
GDP	GDP per capita	thousand constant 2005 US$	World Development Indicators (WDI)
Trade Openness	Sum of exports and imports of goods and services as a percent of GDP	% of GDP	World Development Indicators (WDI)
Exports	Bilateral export values as a percent of total exports	% of total exports of goods and services	World Integrated Trade Solutions (WITS)
Magnitude	Measure of seismic energy		U.S. Geological Survey (USGS)
Tsunami	Indicator variable that takes the value of one when a tsunami was generated by an earthquake		National Oceanic and Atmospheric Administration (NOAA)
No. of deaths	Total number of deaths from an earthquake and secondary effects	thousands	National Oceanic and Atmospheric Administration (NOAA)
Affected population	Total number of people in a buffer zone of 200 km around earthquake's epicenter (using 1990 population survey)	millions	Author's own calculations
Distance	Geodetic distances between financial markets and earthquake's epicenter	thousand kilometers	Author's own calculations

Financial market data comprise daily series of the broadest stock market price indices from Datastream Global Equity Indices available for 35 financial markets. These are aggregate market indices calculated from data on a representative sample of stocks covering a minimum of 75 to 80 percent of total market capitalization in each market. An advantage of using Datastream Indices as opposed to market specific indices (e.g. S&P 500, Nikkei 225) is that they are calculated using the same methods for all the countries/regions included in the analysis. The data for developed economies start in 1973 but the coverage for many other economies starts in the 1990s. We compute daily percentage changes in the stock market price indices to represent continuously compounded daily return.

Table 4 presents descriptive statistics of the daily returns in the 35 financial markets. As seen in the table, except for Argentina, Greece, and Japan, mean daily returns are positive and close to zero. Based on the standard deviations of the returns, stock markets in Turkey and Venezuela are the most volatile and those in New Zealand and Chile the least volatile. Table 4 also shows average values of the market-specific control variables and the distance of the financial markets to earthquake epicenters. While the average real GDP per capita across all financial markets is $22,504, it is only $6,171 for earthquake countries (Table 5). Average trade openness measure is also higher for financial markets (87.55% of GDP) than it is for earthquake countries (51.30% of GDP). The exports to earthquake countries from financial markets are larger than the imports from earthquake countries, which are close to zero for several of the markets. Table 5 also reports that the average magnitude for the 24 earthquakes considered is 7.3. On average, 34,620 people died as a result of earthquakes, 10.5 million people were affected in a 200 km buffer zone, and 54% of the earthquakes resulted in a tsunami.

**Table 4:** Descriptive Statistics for Financial Markets.

	Daily Returns				Macro Variables (Mean)				
Country	Mean	Std. Dev.	Min	Max	GDP per Capita (thousand $)	Trade Openness (% of GDP)	Exports to Earthquake Countries (% of total exports)	Exports of Earthquake Countries (% of total exports)	Distance (thousand km)
Argentina	-0.012	1.839	-33.650	14.348	4.424	31.317	18.504	0.150	13.244
Australia	0.024	1.416	-15.976	8.378	31.353	39.954	29.163	1.351	10.099
Austria	0.014	1.344	-10.378	10.261	34.887	89.272	4.096	0.073	8.057
Belgium	0.022	1.273	-9.341	9.716	33.698	143.186	3.658	0.628	8.386
Canada	0.032	1.282	-13.536	9.519	32.038	71.895	4.743	0.773	10.009
Chile	0.021	1.153	-10.558	12.014	6.955	62.528	21.322	0.151	13.331
China	0.019	2.001	-14.278	15.713	1.520	48.305	17.846	3.117	6.815
Denmark	0.035	1.318	-13.808	11.163	44.360	84.183	4.209	0.150	8.068
Finland	0.030	1.976	-18.573	14.385	33.232	73.089	11.745	0.119	7.734
France	0.021	1.418	-10.694	10.646	31.912	50.552	6.109	1.467	8.494
Germany	0.021	1.378	-8.621	16.262	32.718	66.522	8.081	2.303	8.269
Greece	-0.003	1.842	-11.122	13.667	19.061	53.577	3.145	0.149	7.979
Hong Kong	0.016	1.563	-13.579	15.561	24.377	317.614	2.875	2.958	6.936
Indonesia	0.006	2.466	-37.867	23.246	1.234	60.749	28.886	0.420	8.140
Ireland	0.021	1.429	-14.552	9.324	38.191	158.568	3.477	0.099	8.620
Italy	0.010	1.551	-10.902	11.256	28.979	49.093	7.134	1.993	8.344
Japan	-0.003	1.396	-8.839	11.533	34.412	22.696	13.569	5.078	7.028
Malaysia	0.008	1.616	-36.769	22.984	5.110	190.052	20.538	0.929	7.614
Mexico	0.026	1.732	-20.678	13.735	7.275	56.734	2.771	0.448	11.074
Netherlands	0.017	1.395	-11.487	10.190	37.015	126.024	3.470	1.656	8.337
New Zealand	0.014	1.162	-12.211	9.261	24.871	59.057	15.946	0.096	11.059
Norway	0.029	1.713	-13.586	13.878	60.387	71.567	3.051	0.081	8.060
Philippines	0.012	1.502	-12.031	19.551	1.139	88.619	17.292	0.447	7.282
Poland	0.002	2.059	-12.425	17.004	7.283	64.075	4.900	0.142	7.869
Portugal	0.009	1.278	-12.830	10.912	17.256	64.878	1.509	0.180	9.169
Singapore	0.014	1.293	-9.546	10.619	25.469	361.556	15.289	1.723	7.721
South Africa	0.024	1.698	-14.485	12.096	5.005	53.318	10.691	0.236	10.278
South Korea	0.015	2.346	-21.657	26.849	15.555	76.579	26.921	2.244	6.849
Spain	0.021	1.484	-9.550	13.235	23.626	53.012	4.173	1.113	8.928
Sweden	0.035	1.780	-10.285	13.329	36.789	82.524	6.191	0.172	7.894
Thailand	0.004	1.967	-17.800	16.353	2.482	115.840	20.888	0.805	7.153
Turkey	0.034	2.991	-26.931	22.166	6.446	46.131	5.735	0.468	7.713
UK	0.016	1.274	-10.390	11.817	34.087	56.777	4.534	1.500	8.502
USA	0.028	1.207	-9.409	10.902	38.912	25.057	10.373	9.866	10.140
Venezuela	0.010	2.603	-72.434	33.770	5.571	49.202	5.223	0.448	11.640
Average	0.017				22.504	87.546	10.516	1.244	8.767

**Table 5:** Descriptive Statistics of Earthquake Countries.

	Mean	Std. Dev.	Min	Max
GDP per Capita (thousand $)	6.171	10.220	0.466	36.473
Trade Openness (% of GDP)	51.297	18.682	16.015	99.208
Magnitude	7.254	0.874	6.000	9.100
No. of Deaths (thousands)	34.620	78.046	0.011	316.000
Affected Population (millions)	10.512	11.849	0.111	41.268
Tsunami	0.542	0.509	0	1.000

## Results

Tables 6–9 present the results from the estimation of the GARCH-X(1,1) system of eqs (1a)–(1c) for daily returns. Note that for Venezuela, the GARCH-X(1,1) model failed to achieve convergence; instead a standard GARCH(1,1) model without the earthquake dummies in the conditional variance equation is fit. The top part of Tables 6–9 show the results of the estimation of the conditional mean eq (1a), while the bottom part of the table shows results of the estimation of the conditional variance eq (1c). In the tables, each of the columns refers to one of the 35 financial markets. The first row shows that the marginal effect of one additional earthquake on aggregate stock market returns is not statistically different from zero across all the financial markets except for Malaysia, where the impact on returns is slightly positive (estimated at 0.36 percentage points). This result is broadly consistent with the macroeconomic studies that find an insignificant effect of geological disasters on GDP per capita and its growth [6,10].

**Table 6:** GARCH Estimation Results.

	Argentina	Australia	Austria	Belgium	Canada	Chile	China	Denmark	Finland	France
**Mean Equation**										
Marginal Effects of Earthquakes	0.204	0.206	0.034	-0.003	0.186	-1.093	1.152	-0.173	-0.281	0.078
	(0.281)	(0.200)	(0.191)	(0.163)	(0.192)	(1.264)	(0.965)	(0.206)	(0.264)	(0.207)
d_jt_	0.156	-2.672	2.627	-2.986	0.111	-2.848	12.154*	-0.261	1.341	-6.013
	(5.406)	(5.010)	(4.702)	(2.648)	(4.905)	(5.338)	(5.344)	(3.880)	(3.125)	(5.134)
d_it_						-1.184	0.695			
						(1.234)	(0.987)			
GDP_i_*d_jt_	-0.116	0.027	-0.225	0.012	0.066	-0.570*	-1.851*	-0.067	-0.017	0.380
	(0.440)	(0.122)	(0.189)	(0.101)	(0.047)	(0.278)	(0.761)	(0.114)	(0.082)	(0.298)
GDP_j_*d_jt_	-0.023	-0.017	-0.010	0.006	-0.023*	-0.012	0.012	0.024	0.029	-0.009
	(0.018)	(0.021)	(0.018)	(0.016)	(0.012)	(0.020)	(0.027)	(0.017)	(0.023)	(0.017)
Exports_ij_*d_jt_	0.026	-0.018	0.040	-0.032	-0.118	0.054	-0.362**	-0.068	0.017	-0.278
	(0.160)	(0.054)	(0.214)	(0.056)	(0.199)	(0.041)	(0.126)	(0.420)	(0.164)	(0.332)
Exports_ji_*d_jt_	-0.168	0.031	0.919	-0.009	-0.139	0.060	0.039	1.493	0.867	-0.015
	(1.009)	(0.071)	(1.202)	(0.186)	(0.103)	(0.881)	(0.083)	(0.948)	(1.424)	(0.124)
Trade Openness_i_*d_jt_	0.011	0.058	0.066	0.005	-0.028	0.041	0.053	0.025	0.002	-0.129
	(0.052)	(0.089)	(0.047)	(0.018)	(0.026)	(0.029)	(0.035)	(0.032)	(0.052)	(0.081)
Trade Openness_j_*d_jt_	-0.014	-0.011	0.000	0.003	-0.009	0.000	-0.011	0.025*	0.030	0.003
	(0.009)	(0.010)	(0.011)	(0.010)	(0.008)	(0.008)	(0.012)	(0.012)	(0.017)	(0.011)
Magnitude*d_jt_	0.091	0.096	-0.116	0.317	0.170	0.518	-0.778	0.140	-0.340	0.352
	(0.696)	(0.542)	(0.486)	(0.412)	(0.517)	(0.704)	(0.430)	(0.426)	(0.428)	(0.442)
No. of Deaths*d_jt_	-0.001	-0.002	-0.001	-0.001	-0.003	-0.001	-0.002	0.000	0.000	-0.003
	(0.002)	(0.001)	(0.002)	(0.002)	(0.002)	(0.002)	(0.003)	(0.002)	(0.003)	(0.002)
Affected Population*d_jt_	-0.005	0.010	-0.010	-0.005	-0.006	-0.002	-0.036	0.000	0.008	-0.012
	(0.013)	(0.016)	(0.015)	(0.009)	(0.009)	(0.010)	(0.024)	(0.011)	(0.016)	(0.012)
Distance*d_jt_	0.075	0.035	-0.041	0.165	0.113	0.145	-0.105	-0.224	-0.421	0.192
	(0.295)	(0.330)	(0.312)	(0.277)	(0.323)	(0.332)	(0.302)	(0.298)	(0.312)	(0.288)
Distance*Magnitude*d_jt_	-0.015	-0.008	0.008	-0.031	-0.015	-0.025	0.023	0.016	0.053	-0.031
	(0.040)	(0.045)	(0.047)	(0.039)	(0.045)	(0.047)	(0.040)	(0.041)	(0.043)	(0.042)
Tsunami*d_jt_	0.475	0.346	0.005	0.008	0.227	0.120	1.105**	-0.590	-0.559	0.049
	(0.513)	(0.388)	(0.345)	(0.299)	(0.351)	(0.316)	(0.397)	(0.374)	(0.534)	(0.366)
Return_t-1_	0.097***	0.027	0.039**	0.033*	0.098***	0.179***	0.098***	0.018	0.010	-0.004
	(0.014)	(0.014)	(0.014)	(0.014)	(0.015)	(0.015)	(0.014)	(0.014)	(0.014)	(0.015)
Return_t-2_	-0.011	-0.033*	0.016	-0.007	-0.030*	-0.013	-0.015	-0.019	-0.003	-0.008
	(0.014)	(0.015)	(0.014)	(0.014)	(0.014)	(0.015)	(0.014)	(0.014)	(0.014)	(0.014)
Return_t-3_	0.013	-0.010	-0.014	-0.016	-0.006	-0.032*	0.004	-0.010	-0.033*	-0.045**
	(0.014)	(0.014)	(0.014)	(0.015)	(0.014)	(0.015)	(0.014)	(0.014)	(0.014)	(0.014)
Return_t-4_	0.009	-0.027	-0.006	-0.029*	-0.018	0.028	0.001	-0.016	-0.012	-0.012
	(0.014)	(0.014)	(0.014)	(0.014)	(0.014)	(0.015)	(0.014)	(0.014)	(0.014)	(0.014)
Return_t-5_	-0.022	-0.015	-0.025	-0.021	-0.041**	0.011	-0.012	-0.017	-0.044**	-0.048***
	(0.013)	(0.014)	(0.014)	(0.014)	(0.014)	(0.014)	(0.014)	(0.014)	(0.014)	(0.014)
Year 1997	0.166	0.027	0.035	0.077	0.110	-0.168*	0.292*	0.176*	0.066	0.139
	(0.111)	(0.089)	(0.075)	(0.068)	(0.068)	(0.081)	(0.142)	(0.080)	(0.111)	(0.085)
Year 2008	-0.103	-0.173	-0.105	-0.215*	-0.070	-0.261**	-0.039	-0.121	-0.428**	-0.112
	(0.111)	(0.125)	(0.104)	(0.101)	(0.097)	(0.093)	(0.168)	(0.106)	(0.140)	(0.102)
Constant	0.019	0.003	-0.014	-0.001	-0.006	0.176**	-0.149	-0.048	0.079	-0.064
	(0.088)	(0.066)	(0.055)	(0.050)	(0.050)	(0.063)	(0.096)	(0.057)	(0.084)	(0.061)
**Variance Equation**										
Marginal Effects of Earthquakes	-0.015	0.170	0.025	0.043	0.100	-0.010	0.640	0.081	0.222	0.081
	(0.046)	(0.041)	(0.036)	(0.049)	(0.068)	(0.117)	(1.126)	(0.065)	(0.142)	(0.072)
ARCH	0.115***	0.069***	0.064***	0.081***	0.075***	0.122***	0.104***	0.070***	0.057***	0.071***
	(0.013)	(0.007)	(0.007)	(0.008)	(0.007)	(0.012)	(0.010)	(0.007)	(0.006)	(0.007)
GARCH	0.862***	0.917***	0.929***	0.914***	0.920***	0.843***	0.888***	0.926***	0.940***	0.922***
	(0.013)	(0.009)	(0.007)	(0.007)	(0.007)	(0.014)	(0.009)	(0.007)	(0.006)	(0.007)
Constant	-2.352***	-3.799***	-4.456***	-4.608***	-4.771***	-3.15***	-3.033***	-4.619***	-4.308***	-4.4***
	(0.171)	(0.222)	(0.261)	(0.265)	(0.282)	(0.166)	(0.207)	(0.299)	(0.335)	(0.274)
d_jt_	-0.201	0.478	0.892	1.249	1.854***	0.085	0.727	1.622**	2.073***	1.492*
	(0.728)	(0.774)	(0.713)	(0.673)	(0.503)	(0.580)	(0.756)	(0.557)	(0.538)	(0.583)
d_it_						-0.417	1.203			
						(5.734)	(1.367)			

Sample period is 03/02/1994–08/08/2013. Number of observations per country is 5,072. Standard errors of coefficient estimates are given in parentheses. All equations include year dummy variables from 1995 to 2013, with 1994 as base year. Marginal effects of earthquakes are computed at the mean values of control variables except for dummy variables. Own country earthquake dummy and tsunami dummy variables are set to one. The symbols *, **, and *** indicate statistical significance at the 10%, 5%, and 1% level, respectively.

**Table 7:** GARCH Estimation Results.

	Germany	Greece	Hong Kong	Indonesia	Ireland	Italy	Japan	Malaysia	Mexico	Netherlands
**Mean Equation**										
Marginal Effects of Earthquakes	0.115	-0.399	0.226	1.025	0.009	0.337	-1.228	0.359***	0.229	0.054
	(0.206)	(0.266)	(0.204)	(0.671)	(0.229)	(0.266)	(0.929)	(0.111)	(0.239)	(0.187)
d_jt_	-19.594	-3.349	2.101	0.565	-0.253	-1.621	-0.946	-1.057	-3.558	-1.146
	(18.558)	(5.591)	(3.700)	(3.945)	(3.752)	(6.182)	(13.175)	(1.678)	(5.007)	(2.861)
d_it_				0.782			-1.246			
				(0.783)			(1.027)			
GDP_i_*d_jt_	0.873	0.008	0.010	-2.059	-0.012	0.081	0.011	-0.625	0.484	0.155
	(0.747)	(0.092)	(0.195)	(1.465)	(0.022)	(0.309)	(0.489)	(0.488)	(0.333)	(0.115)
GDP_j_*d_jt_	-0.008	0.053	-0.026	-0.043	-0.002	-0.021	0.022	-0.017	-0.030	0.006
	(0.019)	(0.027)	(0.017)	(0.024)	(0.018)	(0.019)	(0.024)	(0.010)	(0.016)	(0.016)
Exports_ij_*d_jt_	0.021	-0.677	-0.252	0.098	-0.425	0.090	-0.109	0.183	0.027	0.039
	(0.149)	(0.481)	(0.165)	(0.116)	(0.455)	(0.104)	(0.058)	(0.117)	(0.207)	(0.176)
Exports_ji_*d_jt_	-0.024	-1.719	-0.047	1.155*	-0.433	0.034	-0.010	-0.082	-0.054	-0.032
	(0.080)	(1.352)	(0.048)	(0.530)	(1.740)	(0.049)	(0.022)	(0.096)	(0.410)	(0.106)
Trade Openness_i_*d_jt_	-0.130	-0.028	-0.005	0.016	0.000	-0.016	0.080	0.022**	-0.007	-0.044
	(0.122)	(0.034)	(0.013)	(0.022)	(0.008)	(0.078)	(0.137)	(0.008)	(0.017)	(0.027)
Trade Openness_j_*d_jt_	0.004	0.027	0.001	0.010	0.003	-0.016	0.007	-0.001	-0.005	0.003
	(0.012)	(0.017)	(0.010)	(0.012)	(0.012)	(0.012)	(0.011)	(0.006)	(0.010)	(0.010)
Magnitude*d_jt_	-0.101	0.975	0.015	-0.397	0.309	-0.012	-0.026	-0.469*	0.119	0.123
	(0.496)	(0.798)	(0.322)	(0.399)	(0.455)	(0.501)	(0.380)	(0.201)	(0.560)	(0.409)
No. of Deaths*d_jt_	-0.002	-0.003	-0.003	0.000	-0.002	-0.003	0.003	-0.001	-0.003	-0.003
	(0.002)	(0.003)	(0.002)	(0.002)	(0.002)	(0.002)	(0.002)	(0.001)	(0.002)	(0.002)
Affected Population*d_jt_	0.011	-0.002	0.006	-0.014	0.000	-0.001	-0.003	-0.013	-0.005	-0.003
	(0.014)	(0.018)	(0.014)	(0.017)	(0.016)	(0.012)	(0.014)	(0.009)	(0.014)	(0.010)
Distance*d_jt_	-0.128	0.082	-0.002	-0.234	0.127	0.053	-0.225	-0.356**	0.039	0.016
	(0.302)	(0.525)	(0.193)	(0.297)	(0.288)	(0.335)	(0.304)	(0.135)	(0.277)	(0.276)
Distance*Magnitude*d_jt_	0.017	-0.030	-0.001	0.038	-0.019	-0.002	0.035	0.044*	-0.005	-0.004
	(0.046)	(0.079)	(0.026)	(0.039)	(0.042)	(0.049)	(0.040)	(0.018)	(0.040)	(0.041)
Tsunami*d_jt_	0.162	-0.927*	0.511	0.462	-0.011	0.515	-0.275	0.681**	0.305	0.067
	(0.363)	(0.466)	(0.427)	(0.445)	(0.413)	(0.473)	(0.493)	(0.214)	(0.396)	(0.336)
Return_t-1_	-0.003	0.085***	0.034*	0.087***	0.036*	0.004	-0.037*	0.100***	0.133***	0.005
	(0.015)	(0.014)	(0.014)	(0.014)	(0.014)	(0.015)	(0.015)	(0.014)	(0.015)	(0.015)
Return_t-2_	-0.003	-0.027*	-0.014	-0.011	-0.008	0.002	-0.025	0.001	-0.040**	-0.016
	(0.014)	(0.014)	(0.014)	(0.014)	(0.014)	(0.014)	(0.014)	(0.014)	(0.015)	(0.014)
Return_t-3_	-0.033*	-0.005	0.013	-0.013	-0.029*	-0.016	-0.029*	0.008	-0.012	-0.038**
	(0.014)	(0.014)	(0.014)	(0.014)	(0.014)	(0.014)	(0.014)	(0.014)	(0.014)	(0.014)
Return_t-4_	0.002	-0.008	-0.015	-0.018	-0.020	0.007	-0.024	-0.022	-0.017	-0.005
	(0.014)	(0.014)	(0.014)	(0.014)	(0.014)	(0.014)	(0.014)	(0.014)	(0.014)	(0.014)
Return_t-5_	-0.029*	-0.013	-0.016	0.000	-0.021	-0.036*	-0.033*	-0.001	-0.006	-0.033*
	(0.014)	(0.014)	(0.014)	(0.014)	(0.014)	(0.014)	(0.015)	(0.014)	(0.014)	(0.014)
Year 1997	0.072	0.222	0.162	0.074	0.081	0.147	-0.101	-0.053	0.289**	0.103
	(0.082)	(0.114)	(0.116)	(0.097)	(0.076)	(0.116)	(0.099)	(0.087)	(0.110)	(0.079)
Year 2008	-0.225*	-0.178	-0.117	-0.069	-0.307*	-0.186	-0.135	-0.141	-0.045	-0.200*
	(0.093)	(0.123)	(0.132)	(0.115)	(0.123)	(0.124)	(0.109)	(0.091)	(0.116)	(0.100)
Constant	0.046	-0.030	-0.080	-0.031	0.018	-0.019	0.006	-0.029	-0.055	0.026
	(0.058)	(0.077)	(0.088)	(0.066)	(0.057)	(0.093)	(0.064)	(0.066)	(0.089)	(0.052)
**Variance Equation**										
Marginal Effects of Earthquakes	0.066	0.134	0.066	0.149	0.080	0.010	2.071*	0.043	-0.004	0.064
	(0.640)	(0.145)	(0.078)	(0.583)	(0.068)	(0.042)	(1.119)	(0.048)	(0.032)	(0.064)
ARCH	0.076***	0.093***	0.067***	0.145***	0.070***	0.085***	0.077***	0.118***	0.098***	0.079***
	(0.007)	(0.009)	(0.007)	(0.014)	(0.007)	(0.008)	(0.008)	(0.012)	(0.010)	(0.007)
GARCH	0.919***	0.902***	0.929***	0.861***	0.925***	0.910***	0.909***	0.881***	0.886***	0.916***
	(0.008)	(0.008)	(0.007)	(0.010)	(0.007)	(0.008)	(0.009)	(0.009)	(0.010)	(0.007)
Constant	-4.453***	-3.532***	-4.333***	-2.814***	-4.474***	-4.017***	-3.577***	-4.037***	-3.034***	-4.567***
	(0.271)	(0.226)	(0.265)	(0.187)	(0.267)	(0.252)	(0.225)	(0.188)	(0.187)	(0.269)
d_jt_	1.400*	1.277*	1.328	0.392	1.528**	0.387	0.376	0.952	-0.087	1.445*
	(0.612)	(0.633)	(0.704)	(0.953)	(0.565)	(1.164)	(0.985)	(0.555)	(0.788)	(0.636)
d_it_				0.562			2.779**			
				(2.122)			(1.073)			

Sample period is 03/02/1994–08/08/2013. Number of observations per country is 5,072. Standard errors of coefficient estimates are given in parentheses. All equations include year dummy variables from 1995 to 2013, with 1994 as base year. Marginal effects of earthquakes are computed at the mean values of control variables except for dummy variables. Own country earthquake dummy and tsunami dummy variables are set to one. The symbols *, **, and *** indicate statistical significance at the 10%, 5%, and 1% level, respectively.

**Table 8:** GARCH Estimation Results.

	New Zealand	Norway	Philippines	Poland	Portugal	Singapore	South Africa	South Korea	Spain	Sweden
**Mean Equation**										
Marginal Effects of Earthquakes	0.893	0.227	0.271	0.063	0.152	0.060	-0.280	0.228	0.130	-0.020
	(1.108)	(0.233)	(0.179)	(0.344)	(0.193)	(0.160)	(0.196)	(0.277)	(0.203)	(0.287)
d_jt_	-7.730	-1.541	2.724	-7.198	-0.814	-1.654	-15.624	-1.587	-2.867	3.242
	(5.763)	(4.994)	(2.468)	(7.213)	(3.535)	(2.226)	(10.482)	(3.235)	(3.123)	(3.736)
d_it_	0.774									
	(1.179)									
GDP_i_*d_jt_	0.033	0.055	-1.753	0.400	0.289	0.031	2.998	0.190	0.095	0.015
	(0.068)	(0.031)	(1.521)	(0.705)	(0.162)	(0.063)	(2.863)	(0.214)	(0.102)	(0.105)
GDP_j_*d_jt_	-0.021	-0.006	-0.026	-0.021	-0.022	-0.012	-0.006	-0.005	0.008	0.011
	(0.017)	(0.022)	(0.014)	(0.030)	(0.017)	(0.014)	(0.022)	(0.024)	(0.016)	(0.025)
Exports_ij_*d_jt_	0.023	-0.174	0.070	-0.066	0.228	-0.058	-0.272	-0.028	-0.171	-0.334
	(0.096)	(0.402)	(0.044)	(0.141)	(0.415)	(0.050)	(0.280)	(0.110)	(0.297)	(0.251)
Exports_ji_*d_jt_	0.299	1.261	0.267	-1.626	-0.011	-0.064	-0.230	-0.068	-0.030	0.386
	(1.692)	(2.572)	(0.275)	(1.369)	(0.371)	(0.058)	(0.885)	(0.100)	(0.062)	(1.316)
Trade Openness_i_*d_jt_	0.036	-0.047	-0.010	-0.024	-0.042	0.009	-0.013	-0.043*	-0.021	0.005
	(0.024)	(0.069)	(0.011)	(0.082)	(0.042)	(0.006)	(0.049)	(0.021)	(0.035)	(0.045)
Trade Openness_j_*d_jt_	-0.011	0.003	-0.004	0.003	-0.013	0.005	0.001	0.004	0.005	0.012
	(0.014)	(0.014)	(0.009)	(0.019)	(0.011)	(0.008)	(0.015)	(0.016)	(0.012)	(0.016)
Magnitude*d_jt_	0.701	0.321	-0.137	0.934	-0.197	-0.175	0.651	0.323	0.372	-0.336
	(0.606)	(0.510)	(0.317)	(1.018)	(0.506)	(0.214)	(0.784)	(0.398)	(0.451)	(0.519)
No. of Deaths*d_jt_	-0.001	-0.004	-0.002	-0.003	-0.005**	0.001	-0.001	-0.002	-0.003	-0.001
	(0.002)	(0.002)	(0.001)	(0.003)	(0.002)	(0.002)	(0.002)	(0.003)	(0.002)	(0.003)
Affected Population*d_jt_	0.004	-0.006	-0.009	0.000	-0.009	-0.012	0.000	0.005	0.001	-0.014
	(0.012)	(0.021)	(0.011)	(0.025)	(0.010)	(0.010)	(0.017)	(0.014)	(0.012)	(0.019)
Distance*d_jt_	0.504	0.257	-0.036	0.467	-0.104	-0.237	0.188	0.071	0.128	-0.283
	(0.378)	(0.391)	(0.229)	(0.537)	(0.343)	(0.151)	(0.550)	(0.396)	(0.303)	(0.370)
Distance*Magnitude*d_jt_	-0.072	-0.038	0.005	-0.071	0.023	0.027	-0.028	-0.003	-0.024	0.038
	(0.051)	(0.054)	(0.029)	(0.080)	(0.048)	(0.021)	(0.072)	(0.049)	(0.044)	(0.054)
Tsunami*d_jt_	0.304	0.142	0.651	0.161	0.292	0.315	-0.599	0.244	0.113	-0.244
	(0.365)	(0.413)	(0.338)	(0.623)	(0.402)	(0.290)	(0.398)	(0.495)	(0.394)	(0.528)
Return_t-1_	0.035*	0.015	0.156***	0.094***	0.070***	0.036*	0.079***	0.036*	0.034*	0.033*
	(0.014)	(0.014)	(0.015)	(0.014)	(0.014)	(0.015)	(0.015)	(0.015)	(0.015)	(0.015)
Return_t-2_	-0.014	-0.007	-0.008	-0.011	0.013	-0.005	-0.012	-0.016	-0.016	-0.018
	(0.014)	(0.014)	(0.015)	(0.014)	(0.014)	(0.014)	(0.014)	(0.014)	(0.014)	(0.015)
Return_t-3_	-0.019	-0.027	-0.027	-0.012	-0.012	-0.010	-0.011	-0.001	-0.016	-0.029*
	(0.014)	(0.014)	(0.014)	(0.014)	(0.014)	(0.014)	(0.014)	(0.014)	(0.014)	(0.014)
Return_t-4_	-0.017	-0.002	0.004	-0.005	0.013	0.004	-0.026	-0.030*	-0.008	-0.015
	(0.014)	(0.014)	(0.014)	(0.014)	(0.014)	(0.014)	(0.014)	(0.014)	(0.014)	(0.015)
Return_t-5_	0.006	-0.021	-0.026	-0.013	-0.025	0.008	-0.013	-0.024	-0.024	-0.051***
	(0.014)	(0.014)	(0.014)	(0.014)	(0.014)	(0.014)	(0.015)	(0.014)	(0.014)	(0.015)
Year 1997	0.002	0.067	-0.136	0.500*	0.139	-0.091	-0.032	-0.085	0.179	0.134
	(0.086)	(0.092)	(0.107)	(0.219)	(0.081)	(0.076)	(0.086)	(0.126)	(0.097)	(0.103)
Year 2008	-0.163	-0.169	-0.256*	0.305	-0.204*	-0.208*	-0.260*	-0.275*	-0.149	-0.150
	(0.100)	(0.138)	(0.105)	(0.227)	(0.101)	(0.091)	(0.130)	(0.138)	(0.111)	(0.130)
Constant	0.008	0.048	0.097	-0.442*	-0.022	0.021	0.109	0.004	-0.065	-0.018
	(0.068)	(0.067)	(0.073)	(0.198)	(0.058)	(0.052)	(0.067)	(0.082)	(0.072)	(0.079)
**Variance Equation**										
Marginal Effects of Earthquakes	0.000	0.115	-0.023	0.163	0.032	0.060	0.031	0.030	0.057	0.056
	(0.000)	(0.104)	(0.037)	(0.152)	(0.047)	(0.062)	(0.066)	(0.097)	(0.069)	(0.062)
ARCH	0.049***	0.074***	0.170***	0.078***	0.069***	0.092***	0.097***	0.071***	0.072***	0.071***
	(0.006)	(0.007)	(0.016)	(0.008)	(0.007)	(0.009)	(0.009)	(0.007)	(0.007)	(0.005)
GARCH	0.939***	0.919***	0.800***	0.909***	0.928***	0.901***	0.901***	0.924***	0.924***	0.922***
	(0.007)	(0.007)	(0.016)	(0.009)	(0.007)	(0.009)	(0.008)	(0.007)	(0.007)	(0.005)
Constant	-4.221***	-4.004***	-2.446***	-2.961***	-4.827***	-4.252***	-3.900***	-3.498***	-4.390***	-3.871***
	(0.243)	(0.260)	(0.151)	(0.222)	(0.285)	(0.231)	(0.275)	(0.243)	(0.279)	(0.194)
d_jt_	0.899	1.463*	-2.032	1.075*	1.2	1.235*	0.732	0.569	1.278	0.995
	(0.639)	(0.577)	(3.135)	(0.510)	(0.840)	(0.599)	(0.941)	(1.170)	(0.722)	(0.580)
d_it_	-49.395***									
	(2.606)									

Sample period is 03/02/1994–08/08/2013. Number of observations per country is 5,072. Standard errors of coefficient estimates are given in parentheses. All equations include year dummy variables from 1995 to 2013, with 1994 as base year. Marginal effects of earthquakes are computed at the mean values of control variables except for dummy variables. Own country earthquake dummy and tsunami dummy variables are set to one. The symbols *, **, and *** indicate statistical significance at the 10%, 5%, and 1% level, respectively.

**Table 9:** GARCH Estimation Results.

	Thailand	Turkey	UK	USA	Venezuela
**Mean Equation**					
Marginal Effects of Earthquakes	0.290	-2.003	0.143	0.203	0.751
	(0.213)	(5.709)	(0.182)	(0.149)	(1.023)
d_jt_	3.812	6.971	-1.014	-3.772	14.538
	(2.928)	(5.503)	(3.641)	(3.054)	(18.208)
d_it_		-2.371			
		(5.772)			
GDP_i_*d_jt_	-2.124	0.507	0.063	0.174*	-0.681
	(1.284)	(0.435)	(0.051)	(0.085)	(1.194)
GDP_j_*d_jt_	0.012	0.030	-0.017	-0.024*	-0.018
	(0.030)	(0.036)	(0.017)	(0.010)	(0.085)
Exports_ij_*d_jt_	0.200	-0.242	0.104	0.289	-0.057
	(0.239)	(0.255)	(0.373)	(0.182)	(0.222)
Exports_ji_*d_jt_	-0.082	0.068	-0.144	-0.012	-0.065
	(0.260)	(0.274)	(0.115)	(0.018)	(0.481)
Trade Openness_i_*d_jt_	0.025	-0.120*	-0.056	-0.164	-0.072
	(0.018)	(0.061)	(0.066)	(0.088)	(0.086)
Trade Openness_j_*d_jt_	-0.005	0.025	-0.010	-0.010	-0.012
	(0.013)	(0.025)	(0.011)	(0.007)	(0.047)
Magnitude*d_jt_	-0.753*	-0.502	0.361	-0.144	-0.881
	(0.347)	(0.748)	(0.432)	(0.479)	(2.696)
No. of Deaths*d_jt_	-0.001	0.001	-0.003	-0.003*	-0.005
	(0.003)	(0.003)	(0.002)	(0.002)	(0.013)
Affected Population*d_jt_	-0.021	-0.019	-0.004	0.007	0.012
	(0.013)	(0.029)	(0.012)	(0.011)	(0.047)
Distance*d_jt_	-0.296	-0.647	0.253	-0.108	-0.314
	(0.274)	(0.504)	(0.277)	(0.305)	(1.473)
Distance*Magnitude*d_jt_	0.035	0.066	-0.039	0.010	0.040
	(0.035)	(0.075)	(0.041)	(0.042)	(0.202)
Tsunami*d_jt_	0.978*	0.634	0.294	0.495	1.327
	(0.432)	(0.810)	(0.337)	(0.279)	(1.826)
Return_t-1_	0.053***	0.040**	-0.011	-0.022	0.094***
	(0.014)	(0.014)	(0.015)	(0.015)	(0.024)
Return_t-2_	0.034*	-0.007	-0.024	-0.034*	-0.022
	(0.014)	(0.014)	(0.014)	(0.014)	(0.021)
Return_t-3_	-0.014	-0.009	-0.046**	-0.045**	-0.005
	(0.014)	(0.014)	(0.014)	(0.014)	(0.019)
Return_t-4_	-0.020	0.005	-0.027	-0.028*	0.011
	(0.014)	(0.014)	(0.014)	(0.014)	(0.018)
Return_t-5_	-0.008	-0.023	-0.060***	-0.036**	0.015
	(0.014)	(0.014)	(0.014)	(0.014)	(0.019)
Year 1997	-0.443**	0.183	0.091	0.131	0.281
	(0.156)	(0.226)	(0.075)	(0.069)	(0.334)
Year 2008	-0.199	-0.467*	-0.220*	-0.093	0.107
	(0.131)	(0.221)	(0.103)	(0.087)	(0.597)
Constant	0.066	0.186	-0.012	0.020	-0.167
	(0.091)	(0.170)	(0.055)	(0.045)	(0.179)
**Variance Equation**					
Marginal Effects of Earthquakes	0.257	0.048	0.036	0.084	
	(0.190)	(0.509)	(0.046)	(0.060)	
ARCH	0.104***	0.111***	0.077***	0.081***	0.134***
	(0.010)	(0.010)	(0.007)	(0.008)	(0.015)
GARCH	0.884***	0.879***	0.915***	0.916***	0.155***
	(0.010)	(0.010)	(0.008)	(0.008)	(0.042)
Constant	-2.873***	-1.969***	-4.514***	-4.887***	
	(0.194)	(0.194)	(0.251)	(0.278)	
d_jt_	1.272**	0.695	1.105	1.812***	
	(0.440)	(0.741)	(0.696)	(0.529)	
d_it_		-0.431			
		(2.325)			

Sample period is 03/02/1994–08/08/2013. Number of observations per country is 5,072. Standard errors of coefficient estimates are given in parentheses. All equations include year dummy variables from 1995 to 2013, with 1994 as base year. Marginal effects of earthquakes are computed at the mean values of control variables except for dummy variables. Own country earthquake dummy and tsunami dummy variables are set to one. The symbols *, **, and *** indicate statistical significance at the 10%, 5%, and 1% level, respectively.

The own earthquake dummy (*d*_*it*_) is not statistically significant for any of the financial markets that experienced an earthquake during the sample period. The earthquake dummy (*d*_*jt*_) is also statistically insignificant across financial markets except for China, where the magnitude is large (12.2 percentage points). However, even in this case, the marginal effect of earthquakes on stock market returns, once the interaction with other explanatory variables in eq (1a) is taken into account, is statistically indistinguishable from zero (except, as indicated above, for Malaysia where it is very small).

The estimates for the interactions with macro variables are mostly insignificant (this is the case for 24 out of the 35 financial markets), and differ by financial market, but a few regularities emerge regarding those coefficients that are statistically significant. We hypothesized that, everything else being equal, earthquakes that happen in more developed economies would have a more negative impact on stock returns than earthquakes affecting less developed countries. As expected, this is the case for the markets in Canada and the US. We also hypothesized that financial markets in richer countries would be more resilient to earthquakes as income is widely thought to reduce vulnerability to natural disasters. Consistent with this expectation, *GDP*_*i*_ has a positive sign for the US financial market. It, however, exhibits a negative sign for the financial markets in Chile and China. Bilateral trade flows and openness indicators are mostly insignificant and when they are not, the signs are country-specific. These likely reflect different sectorial composition of trade flows. While for some countries/regions a disruption in supply and/or demand chains caused by earthquakes has negative impacts, for others it may present an opportunity for domestic firms. Similarly, while increased trade openness mitigates the negative impact of earthquakes in the stock markets of Malaysia, it increases the vulnerability in South Korea and Turkey.

Regarding the earthquake characteristics, larger earthquakes in terms of magnitude have a negative impact on returns in the markets of Thailand and Malaysia. In the latter, also as expected, this effect decays with the distance to the epicenter of the earthquake. Another earthquake indicator, the number of deaths, dampens financial returns in Portugal and the US. The tsunami indicator has the expected sign for Greece, but exhibits a positive sign in the Chinese, Malaysian, and Thai markets.

The results indicate that accounting for autocorrelation is important, as illustrated by the statistical significance of multiple lagged returns. In all the financial markets, except for Denmark and Norway, there is at least one lagged return that is statistically significant (typically this is the first one, but significance is observed up to the 5^th^ lag). As expected, the coefficient for the year 2008 dummy variable representing the recent global financial crisis is found to be negative for 33 markets; for 13 markets the estimates are statistically significant at conventional confidence levels. For the Asian financial crisis, represented with the 1997 year dummy, the results are more mixed. While two countries (Chile and Thailand) have negative and significant coefficients, four countries (China, Denmark, Mexico, and Poland) have positive estimates.

The results for the variance equation at the bottom of the table indicate that a GARCH structure for the variance of the error term is appropriate. Both the ARCH and GARCH terms are statistically significant at the 1% level in all the financial markets. Earthquake dummy coefficients in the conditional variance equation are statistically significant for 15 of the 34 financial markets. However, once the derivative of the variance equation with respect to the earthquake dummy is taken, the marginal effect of earthquakes is statistically significant only for Japan.

## Conclusions

We analyze the impacts of extreme earthquakes on the conditional mean and variance of daily returns of 35 aggregate stock market indices over the last twenty years. Overall, the results suggest that financial markets are resilient to earthquake shocks. This is the case for each of the 35 markets considered over a five-day event window following the earthquake shock. The marginal effect of an additional earthquake on financial returns (as measured by aggregate market indices) is zero for the vast majority: 34 out of the 35 financial markets considered. For Malaysia, the marginal effect is slightly positive (0.36 percentage points). This result is robust to accounting for earthquakes happening where the financial market is located. For Japan, we find evidence that domestic earthquakes increased the volatility of its financial returns.

Overall, our results are consistent with studies that have shown that geological disasters have no lasting impacts on the GDP or GDP growth of the countries affected. We note, however, that the aggregate stock market indices capture the performance of the part of the economy represented by publicly traded companies and, as such, our results cannot be directly compared with those assessing overall macroeconomic performance following natural disaster shocks. Moreover, our results are contingent on the characteristics of the historical earthquakes included in the sample. The reaction of the stock markets might be different for a substantially larger earthquake (as unlikely as this may be since we are already considering devastating earthquakes).

Of course, large earthquakes can and do cause immense hardship to individuals and businesses. All the 24 events included in the analysis are “extreme” in terms of casualties (an average of 35,000) or of the immediate economic damages (over 2.5% of GDP). But there are often gainers as well as losers, and our results suggest that the net effect for aggregate financial markets is zero. This result is reminiscent of the finding by Cutler et al. [51] that noneconomic newsworthy events (entries in the “Chronology of Important World Events” from the World Almanac for 1941–1987) had a surprisingly small effect on aggregated stock market returns. A disaggregated analysis of stock market indices for specific sectors such as construction and insurance might, however, tell a different story.

Our results are robust to controlling for the heterogeneity of impacts of the “average” large earthquake. We control for key characteristics of the earthquake (such as its magnitude, fatalities, and population affected) and of the economies included in the analysis (those home of a financial market and those affected by large earthquakes, including the trade linkages between the two). Few of these controls, notably GDP per capita and trade openness among the macro variables; and fatalities, magnitude, distance to the epicenter and whether it resulted in a tsunami among the earthquake characteristics, are found to mediate the impact of earthquakes on abnormal returns, and their influence is market-specific. In any case, the estimated marginal effect of an additional earthquake on financial returns is never negative, and earthquakes increase stock market volatility only in Japan.
